# Serologic Detection of Middle East Respiratory Syndrome Coronavirus Functional Antibodies

**DOI:** 10.3201/eid2605.190921

**Published:** 2020-05

**Authors:** Nisreen M.A. Okba, Ivy Widjaja, Wentao Li, Corine H. GeurtsvanKessel, Elmoubasher A.B.A. Farag, Mohammed Al-Hajri, Wan Beom Park, Myoung-don Oh, Chantal B.E.M. Reusken, Marion P.G. Koopmans, Berend-Jan Bosch, Bart L. Haagmans

**Affiliations:** Erasmus Medical Center, Rotterdam, the Netherlands (N.M.A. Okba, C.H. GeurtsvanKessel, C.B.E.M. Reusken, M.P.G. Koopmans, B.L. Haagmans);; Utrecht University, Utrecht, the Netherlands (I. Widjaja, W. Li, B.-J. Bosch);; Ministry of Public Health, Doha, Qatar (E.A.B.A. Farag, M. Al-Hajri);; Seoul National University College of Medicine, Seoul, South Korea (W.B. Park, M.-d. Oh);; Center for Infectious Disease Control, Bilthoven, the Netherlands (C.B.E.M. Reusken)

**Keywords:** Middle East respiratory syndrome coronavirus, MERS-CoV, neutralization, hemagglutination, lumazine synthase, antibodies, human, dromedary, serology, nanoparticle, spike, viruses, zoonoses, respiratory infections, Middle East respiratory syndrome, MERS

## Abstract

We developed and validated 2 species-independent protein-based assays to detect Middle East respiratory syndrome coronavirus functional antibodies that can block virus receptor-binding or sialic acid-attachment. Antibody levels measured in both assays correlated strongly with virus-neutralizing antibody titers, proving their use for serologic confirmatory diagnosis of Middle East respiratory syndrome.

The zoonotic introductions and ongoing outbreaks of Middle East respiratory syndrome (MERS) coronavirus (MERS-CoV) pose a global threat (*1*,*2*) necessitating continuous serosurveillance to monitor virus spread alongside the development of vaccine and antibodies as countermeasures. Both approaches require validated assays to evaluate specific antibody responses. Although MERS-CoV serologic assays have been developed (*2*–*6*), those detecting functional antibodies cannot be applied in all laboratories and can require Biosafety Level 3 (BSL-3) containment. Recombinant protein-based immunoassays are easier to operate and standardize and do not require BSL-3 containment. However, MERS-CoV protein-based assays developed thus far can only detect antibody binding and give no information on antibody functionality. The MERS-CoV spike protein N terminal subunit (S1) contains 2 functional domains: the N-terminal domain (S1^A^), which binds sialic acid, the viral attachment factor; and the receptor-binding domain (RBD) (S1^B^), which binds dipeptidyl peptidase 4, the virus receptor (*7*,*8*). Antibodies against those 2 domains can block MERS-CoV infection (*9*). Based on this fundamental knowledge, we developed 2 recombinant protein-based functional assays.

First, we developed an S1-based competitive ELISA, a receptor-binding inhibition assay (RBI), to test for antibodies that block the interaction with dipeptidyl peptidase 4, the viral receptor (Appendix Figure 1). We validated the specificity of the assay for human diagnostics using serum samples from healthy blood donors, PCR-confirmed non–coronavirus-infected patients and non–MERS-CoV–infected patients (cohorts H1–H3) (Appendix Table 1). At a 1/20 dilution, none of the samples from non–MERS-CoV-infected humans showed a >50% reduction in signal (RBI_50_) (Figure, panel A), indicating a high specificity of the assay. MERS-CoV–specific RBI antibodies were detected in all the 90% plaque reduction neutralization assay (PRNT_90_)–positive serum samples of the PCR-confirmed MERS-CoV patients tested (Appendix Table 2, Figure 2). The percentage reduction in signal strongly correlated with neutralizing antibody titers (Figure, panel B). The RBI_50_ assay showed similar sensitivity to the PRNT_90_ assay.

**Figure:**
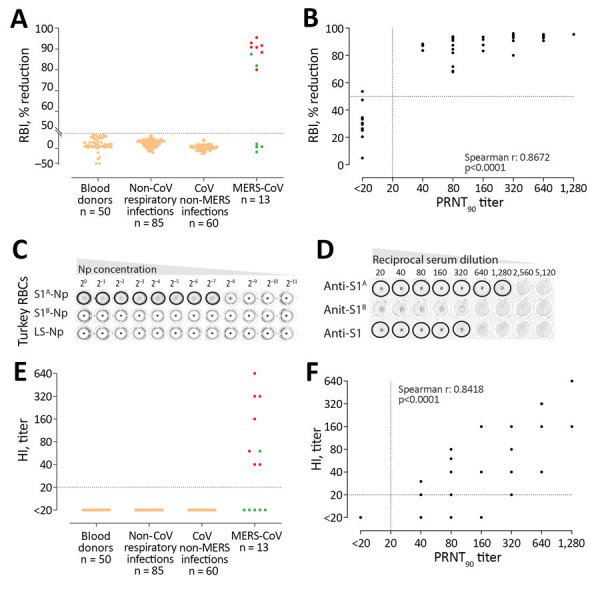
MERS-CoV–specific RBI and HI assays for MERS-CoV human diagnostics. A) Validation of the specificity of the RBI assay for the detection of MERS-CoV–specific antibodies in humans. Red dots indicate severe illness. Green dots indicate mild illness. B) Correlation between neutralizing and RBI antibody responses after MERS-CoV infection. C) Hemagglutination of turkey erythrocytes using S1^A^-nanoparticles. S1^A^-, S1^B^-, or empty self-assembling lumazine synthase nanoparticles were serially diluted and tested for the ablity to agglutinate turkey RBCs. D) Specificity of the HI assay for the detection of MERS-CoV S1^A^–directed antibodies. Rabbit anti-S1^A^, anti S1^B^, or anti-S1 serum samples were serially diluted and tested for the ability to block S1^A^-nanoparticles–induced hemagglutination of turkey RBCs. E) Validation of HI assay for the detection of MERS-CoV–specific antibodies in humans. F) Scatter plot correlating PRNT_90_ neutralization titers and HI titers after MERS-CoV infection. CoV, human coronavirus; HI, hemagglutination inhibition; MERS-CoV, Middle East respiratory syndrome coronavirus; PRNT_90_, 90% reduction in plaque reduction neutralization test; RBI, receptor-binding inhibition.

Because the RBI assay is species-independent, we validated its ability to detect RBI antibodies in dromedaries. At a 1/20 dilution, none of the naive dromedary serum samples (*10*) reacted in the assay, whereas all samples from MERS-CoV–infected dromedaries (*2*) resulted in a >90% reduction in signal (Appendix Table 1, Figure 3, panel A). We detected RBI antibodies in the samples of vaccinated and experimentally infected dromedaries (Appendix Figure 3, panel B). Overall, the RBI_50_ was highly specific and showed comparable sensitivity to PRNT_90_ for detection of MERS-CoV–specific RBI (neutralizing) antibodies after infection and vaccination (Appendix Figure 3, panel C).

Apart from the RBD, the MERS-CoV S1 contains an α2,3 sialic acid–binding S1^A^ domain (*7*). When this domain was multivalently presented on self-assembling lumazine synthase (LS) nanoparticles (S1^A^-Np), it was able to hemagglutinate human erythrocytes. To generate S1^A^-Np, we genetically fused the S1^A^ domain to LS and expressed the particles in HEK-293S cells (Appendix Figure 4, panel A). By using S1^A^-Np, we developed a hemagglutination inhibition (HI) assay to detect antibodies capable of blocking virus interaction with sialic acids (Appendix Figure 4, panel B). To set up the assay using turkey RBCs, we tested the ability of S1^A^-Np to agglutinate turkey erythrocytes by using empty (LS)-Np and S1^B^-Np as negative controls. Although neither the lumazine synthase–Np nor the S1^B^-Np showed any hemagglutination at any temperature tested, the S1^A^-Np induced hemagglutination at 4°C; we also noted hemagglutination when using dromedary erythrocytes (Figure, panel C; Appendix Figure 4, panel C). Although antibodies against the S1 and S1^A^ domain inhibited hemagglutination showing high HI titers, S1^B^ antibodies were negative for HI (Figure, panel D).

Next, we used the same cohort of serum samples for validating the RBI assay. None of the samples from healthy blood donors, PCR-confirmed non–coronavirus-infected and non–MERS-CoV–infected patients (cohorts H1–H3) showed any HI at the 1/20 dilution (Figure, panel E). HI antibodies were detected in the samples of all severely infected MERS-CoV patients and that of 1 mildly infected MERS-CoV patient (Figure, panel E; Appendix Figure 5); only 2 of the mildly infected MERS-CoV patients were PRNT_90_-positive (Appendix Table 2). Serum HI titers correlated strongly with neutralizing antibody titers detected by a whole virus neutralization assay (PRNT_90_); nonetheless, the PRNT_90_ assay was more sensitive (Figure, panel F). Similarly, only serum samples from MERS-CoV–infected dromedaries were HI-positive (10/13), whereas none of the naive dromedary camel serum samples showed any HI (Appendix Figure 6, panel A). HI antibodies were detected in serum samples of vaccinated dromedaries after booster immunization (Appendix Figure 6, panel B). Overall, although less sensitive, the antibody titers detected by the HI assay correlated strongly with the neutralizing antibody titers detected by PRNT_90_ assay (Appendix Figure 6, panel C).

The RBI and HI assays we developed are easy to operate and standardize and can detect functional antibodies against 2 MERS-CoV S1 domains responsible for virus entry (RBD) and attachment (S1^A^). Both assays are protein-based and can be carried out in a 96-well plate format, therefore providing BSL-1 high-throughput platforms. The assays can be used as confirmatory assays for human and dromedary MERS-CoV diagnostics and for antibody and vaccine evaluation.

Appendix Additional information about serologic detection of Middle East respiratory syndrome functional antibodies.
