# Supplementary material for: Serologic Detection of Middle East Respiratory Syndrome Coronavirus Functional Antibodies
Source: Emerg Infect Dis. 2020 May;26(5):1024–7. doi: 10.3201/eid2605.190921 (PMC7181916; doi:10.3201/eid2605.190921)
Supplement: Appendix — Additional information about serologic detection of Middle East respiratory syndrome functional antibodies. [file 19-0921-Techapp-s1.pdf]

Article DOI: https://doi.org/10.3201/eid2605.190921

Serologic Detection of Middle East Respiratory Syndrome Coronavirus Functional Antibodies

Appendix

Materials and Methods

Serum Samples

To validate the specificity of the developed assays for the detection of MERS-CoV-specific antibodies in humans, we used a set of sera we previously described in an earlier study to validate a MERS-CoV specific S1 ELISA (*1*) (Appendix Table 1, cohorts H1–H5). We also included sera from MERS-CoV infected camels (*2*) as well as sera from MERS-CoV vaccinated camels (*3*) to validate the use of the assays for camel MERS-CoV diagnostics and evaluation of immune responses following vaccination (Appendix Table 1, sample sets D1–D3). The use of human sera from the Netherlands was approved by the local medical ethical committee (MEC approval no. 2014–414) and from South Korea by the Institutional Ethics Review Board of Seoul National University Hospital (approval no. 1506–093–681). The use of camel sera was approved as previously described (*2*,*3*)

Protein Production

For the receptor binding inhibition assay, both DPP4 and MERS-S1-mFc were produced in HEK-293T cells as previously described. The DPP4 ectodomain (39–766) was expressed as an N terminally strep-tagged protein and purified from cell culture supernatant using Strep-Tactin sepharose beads (IBA GmbH). MERS-CoV S1 (1–751) was C-terminally fused to a mouse IgG2a Fc in pCAGGS expression vector and purified from cell culture supernatant using protein A sepharose beads.

For the hemagglutination inhibition assay, empty lumazine synthase nanoparticles (LS-Np) were produced as previously described (*4*). $S1^A$ nanoparticles ($S1^A$-Np) were produced by genetically linking the $S1^A$ encoding region of the MERS-CoV spike (amino acids19–357; EMC

strain GenBank Acc. no. JX869059.2) to the lumazine synthase encoding gene in pCAGGS vector encoding a CD5 signal peptide and strep tag. $S1^A$-Np were expressed in HEK-293S cells and purified using Strep-Tactin sepharose beads.

Receptor Binding Inhibition Assay

We developed a competitive ELISA to detect antibodies capable of blocking of the binding of MERS-CoV to its cellular receptor DPP4. ELISA plates were coated overnight at 4°C with 2 ug/ml recombinant DPP4 in PBS. The plates were washed with PBS and blocked with 3% BSA/PBS-0.5% tween-20 for 1 hr at room temp. In the meantime, 1/20 diluted sera (or further 2-fold serially diluted for titer determination) were mixed with 5 ng of S1-mFc in a total volume of 100 ul blocking buffer per well and incubated for 1 hr at room temperature. Wells with no serum (only S1-mFc) were included in each run to calculate to maximum binding. Following 1 hr of incubation the mix was transferred to blocked plates and allowed to incubate for 1 hr further. The plates were washed 3 times with PBS/0.05% tween-20, and the amount of S1-mFc bound to the plate was determined by adding HRP-labeled anti-mouse IgG (1:2000, Dako) and incubating for 1 hr. Following washing, the signal was revealed by adding 100 ul of TMB and the reaction was stopped using sulfuric acid. Absorbance was measured using Tecan. Blocking was determined as percentage reduction of the sample signal from the blank signal (no serum). A 50% reduction in signal (RBI_{50}) in a ≥1/20 diluted sample was considered positive. Serum antibody titers were determined as the reciprocal of the highest serum dilution resulting in a ≥50% signal reduction.

Hemagglutination Assay

We tested the ability of $S1^A$ multivalently-expressed on lumazine synthase nanoparticles ($S1^A$-Np) to agglutinate turkey and dromedary RBCs.

For the HA assay, fifty µl of 2-fold serially diluted Nps were mixed with an equal volume of 0.5% RBCs in PBS and incubated for 1 hr at 4°C. Following incubation, the hemagglutination activity was assessed and the HA titer (HA units; HAU) of the Nps was recorded as the dilution of the last well showing hemagglutination.

Hemagglutination Inhibition Assay

To carry out the hemagglutination inhibition assay, sera were 2-fold serially diluted starting at a 1/10 dilution in a total volume of 50 ul PBS. $S1^A$-Nps corresponding to 4 HAU in 25 ul PBS were added to each well and the mix was incubated for 30 min at 37°C. Following

incubation, 25 ul of 0.5% turkey RBCs in PBS were added and further incubated for 1 hr at 4°C after which the serum HI titer was scored. A serum titer ≥20 was considered positive.

Plaque Reduction Neutralization Assay (PRNT)

All sera included in this study were previously tested for MERS-CoV neutralization using the $PRNT_{90}$ assay (*1*–*3*). Owing to the specificity and sensitivity, neutralization assays are considered the gold standard for MERS-CoV serology. Thus, we compared the performance of the developed assays RBI and HI to $PRNT_{90}$ to assess their specificity and sensitivity.

References

1. Okba NMA, Raj VS, Widjaja I, GeurtsvanKessel CH, de Bruin E, Chandler FD, et al. Sensitive and specific detection of low-level antibody responses in Mild Middle East respiratory syndrome coronavirus infections. Emerg Infect Dis. 2019;25:1868–77. PubMed https://doi.org/10.3201/eid2510.190051

2. Haagmans BL, Al Dhahiry SH, Reusken CB, Raj VS, Galiano M, Myers R, et al. Middle East respiratory syndrome coronavirus in dromedary camels: an outbreak investigation. Lancet Infect Dis. 2014;14:140–5. PubMed https://doi.org/10.1016/S1473-3099(13)70690-X

3. Haagmans BL, van den Brand JM, Raj VS, Volz A, Wohlsein P, Smits SL, et al. An orthopoxvirus-based vaccine reduces virus excretion after MERS-CoV infection in dromedary camels. Science. 2016;351:77–81. PubMed https://doi.org/10.1126/science.aad1283

4. Li W, Hulswit RJG, Widjaja I, Raj VS, McBride R, Peng W, et al. Identification of sialic acid-binding function for the Middle East respiratory syndrome coronavirus spike glycoprotein. Proc Natl Acad Sci U S A. 2017;114:E8508–17. PubMed https://doi.org/10.1073/pnas.1712592114

Appendix Table 1. Sample sets used in this study*

Species	Country	Cohort	Sample source		No. samples	Range (post diagnosis)	Reference
Human	The Netherlands	H1	Blood Donors (Negative cohort)		50	NA	(*1*)
		H2	Non-hCoV Respiratory Infections (N = 85)	Adenovirus	5	2- 4 w	
				Bocavirus	2		
				enterovirus	2		
				HMPV	9		
				Influenza A	13		
				Influenza B	6		
				Rhinovirus	9		
				RSV	9		
				PIV-1	4		
				PIV-3	4		
				M. pneumoniae	1		
				CMV	9		
				EBV	12		
		H3	Recent hCoV infections (N = 60)	α-CoV HCoV-229E	19	>2w –1y	
				HCoV-NL63	18		
				β-CoV HCoV-OC43	23		
		H4†	RT-PCR confirmed MERS cases (n = 60 longitudinal specimen from 13 patients)	Acute	21	1–14 d	
				Convalescent	7	15–228 d	
	South Korea	H5		Mild infection‡	17	6–12 mo	
				Severe infection§	15	6–12 mo	
Dromedary camels	Qatar	D1	MERS-CoV seropositive		13	NA	(*2*)
	Canary Islands	D2	MERS-CoV seronegative (longitudinal specimen)	Vaccinated¶	28	0–63 dpv	(*3*)
		D3	MERS-CoV infected (longitudinal specimen)	Infected#	28	0–14 dpi	

*CoV, coronavirus; CMV, Cytomegalovirus; d, day; dpi, days post-infection; dpv, days post-vaccination; EBV, Epstein-Barr virus; HCoV, human coronavirus; HMPV, Human metapneumovirus; MERS, Middle East respiratory syndrome; mo, month; NA, not applicable; PIV, parainfluenza virus; RSV, respiratory syncytial virus; w, week; y, year.
†Samples taken from 2 case-patients at different time points.
‡Samples taken from 6 case-patients at different time points.
§Samples taken from 5 case-patients at different time points.
¶Samples taken from 4 camels at different time points.
#Samples taken from 4 camels at different time points.

Appendix Table 2. Comparative validation results of the RBI and HI assays versus the $PRNT_{90}$*

					N positives / N tested				
					RBI_{50}		HI		
Species	Cohort	Sample source		No. samples	$PRNT_{90}$ positive	$PRNT_{90}$ negative	$PRNT_{90}$ positive	$PRNT_{90}$ negative	Validation aspect
Human	H1	Blood Donors		50	NA	0/50	NA	0/50	Specificity
	H2	Non-CoV Respiratory Infections (N = 85)	Adenovirus	5	NA	0/5	NA	0/5	
			Bocavirus	2	NA	0/2	NA	0/2	
			Enterovirus	2	NA	0/2	NA	0/2	
			HMPV	9	NA	0/9	NA	0/9	
			Influenza A	13	NA	0/13	NA	0/13	
			Influenza B	6	NA	0/6	NA	0/6	
			Rhinovirus	9	NA	0/9	NA	0/9	
			RSV	9	NA	0/9	NA	0/9	
			PIV-1	4	NA	0/4	NA	0/4	
			PIV-3	4	NA	0/4	NA	0/4	
			M. pneumoniae	1	NA	0/1	NA	0/1	
			CMV	9	NA	0/9	NA	0/9	
			EBV	12	NA	0/12	NA	0/12	
	H3	Recent CoV infections (N = 60)	α-CoV HCoV-229E	19	NA	0/19	NA	0/19	
			HCoV-NL63	18	NA	0/18	NA	0/18	
			β-CoV HCoV-OC43	23	0/2	0/23	0/2	0/23	
	H4†	RT-PCR confirmed	≤14 d post diagnosis	21	11/11	1/10	7/11	0/10	Sensitivity
			>14 d post diagnosis	7	7/7	NA	7/7	NA	
	H5	MERS cases (n = 60 longitudinal specimen from 13 patients)	Mild infection‡	17	5/5	0/12	2/5	0/12	
			Severe infection§	15	15/15	NA	15/15	NA	
Dromedary camels	D1	MERS-CoV seropositive		13	13/13	NA	10/13	NA	
	D2	MERS-CoV seronegative (longitudinal specimen)	Vaccinated¶	28	16/20	0/8	16/20	0/8	
	D3		Infected#	28	5/6	0/22	0/6	0/22	

*CoV, coronavirus; CMV, Cytomegalovirus; EBV, Epstein-Barr virus; HCoV, human coronavirus; HMPV, Human metapneumovirus; MERS, Middle East respiratory syndrome; NA, not applicable; PIV, parainfluenza virus; RSV, respiratory syncytial virus.
†Samples taken from 2 case-patients at different time points.
‡Samples taken from 6 case-patients at different time points.
§Samples taken from 5 case-patients at different time points.
¶Samples taken from 4 camels at different time points.
#Samples taken from 4 camels at different time points.

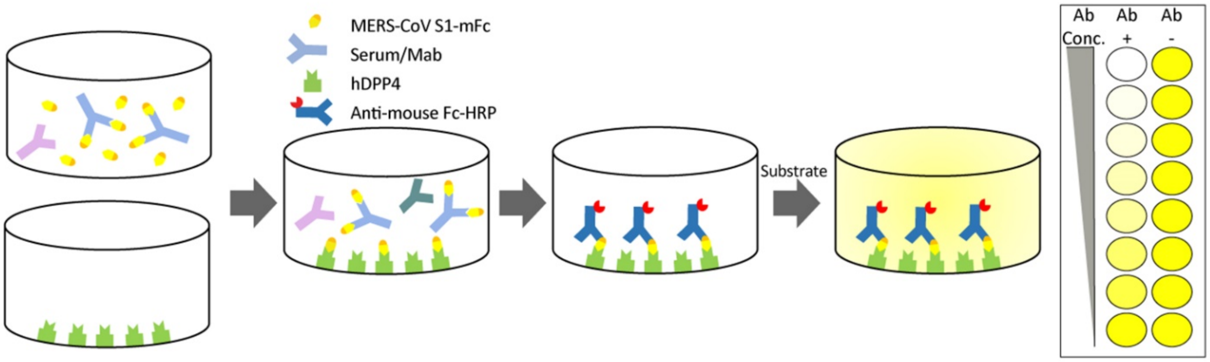

Appendix Figure 1. Schematic diagram showing the principle of the receptor binding inhibition (RBI) assay. S1-mFc, MERS-CoV S1 protein with a mouse Fc tag; hDPP4, human Dipeptidyl Peptidase-4 (MERS-CoV receptor); HRP, horse radish peroxidase.

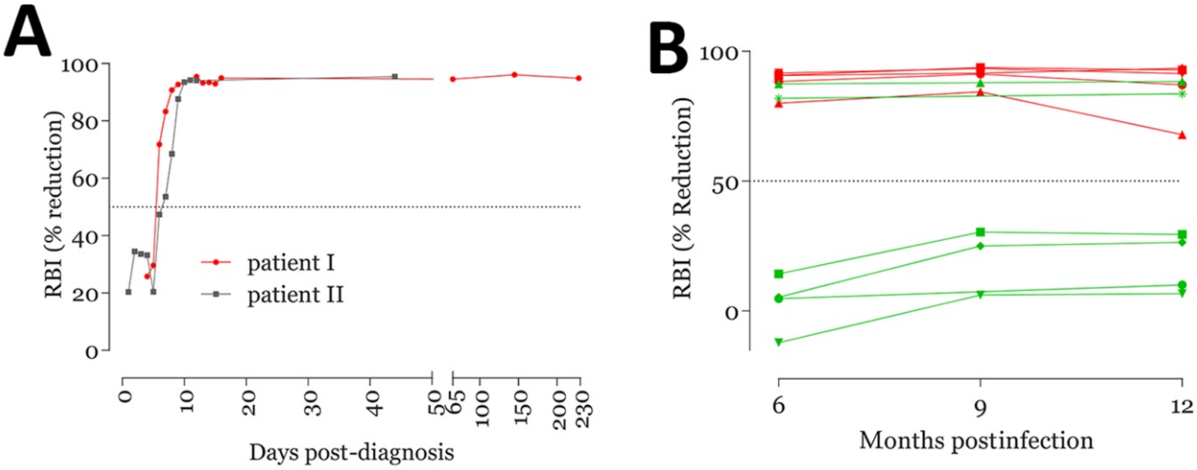

Appendix Figure 2. Kinetics of RBI antibody responses in PCR-confirmed MERS patients. RBI antibody responses in (A) two acute to convalescent phase patients and in (B) severe (red, n = 5) and mild (green, n = 6) MERS-CoV patients six to twelve months post-infection.

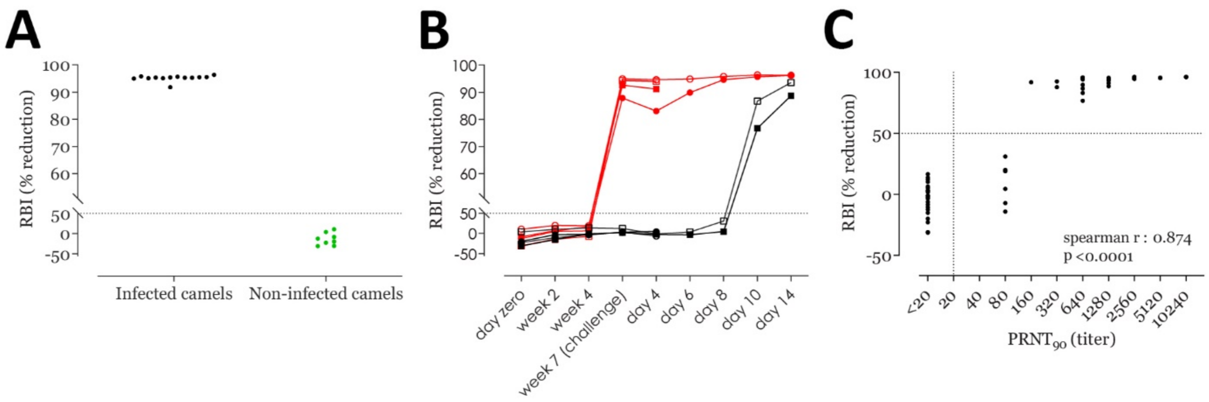

Appendix Figure 3. MERS-CoV specific receptor binding inhibition (RBI) assay for MERS-CoV dromedary camel diagnostics. A)Validation of the specificity of the RBI assay for the detection of MERS-CoV specific antibodies in the sera of MERS-CoV-infected (black) and naïve (green) dromedary camels. B) Kinetics of RBI antibody responses in dromedary camels following vaccination (red) and infection (black). C) Correlation between neutralizing and RBI antibody responses following MERS-CoV infection or vaccination in dromedaries. $PRNT_{90}$, 90% reduction in plaque reduction neutralization test.

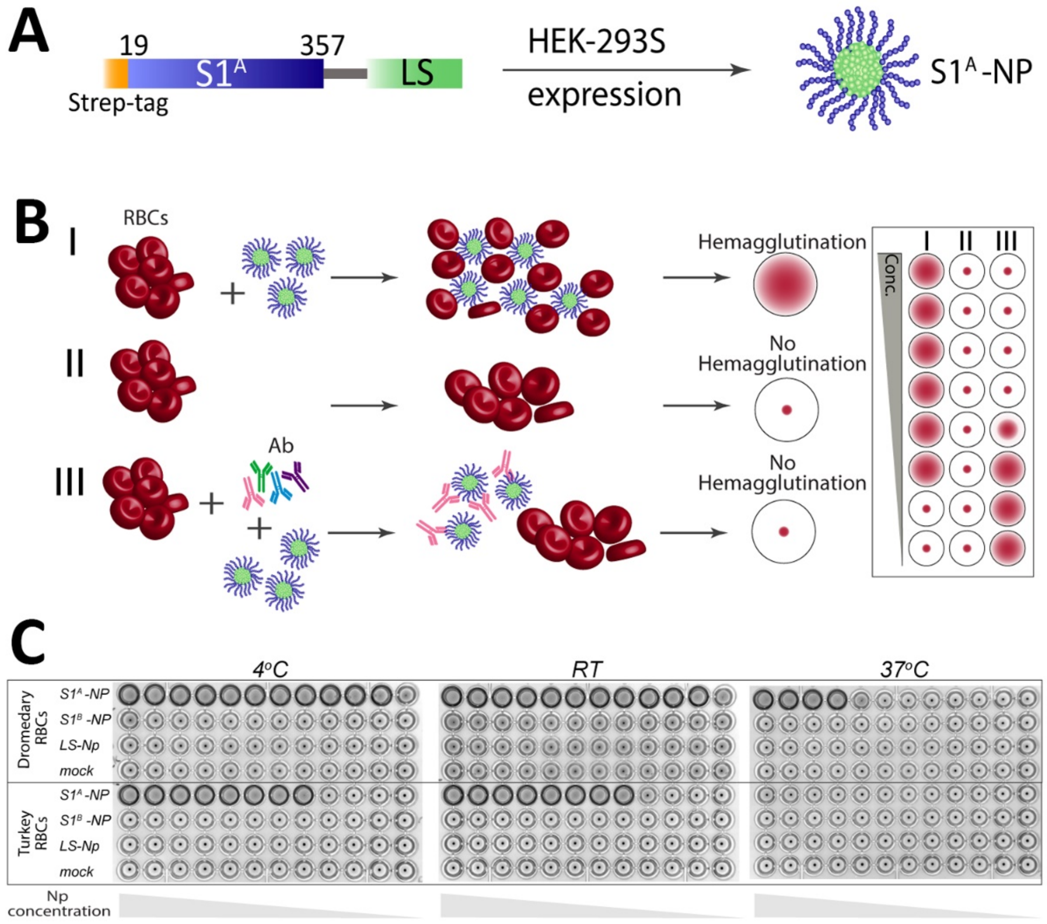

Appendix Figure 4. Development of MERS-CoV HI assay. A) Schematic diagram of the production of S1^{A} lumazine synthase (LS) nanoparticles (Np). B) The principle of the HI assay showing the hemagglutination of red blood cells (RBCs) in the presence of S1^{A}-Np(I), no-hemagglutination in the absence of the particles (II) and the inhibition of hemagglutination (HI) by S1^{A}-directed antibodies (III). C) S1^{A}-Np induced hemagglutination of dromedary and turkey RBCs at different temperatures.

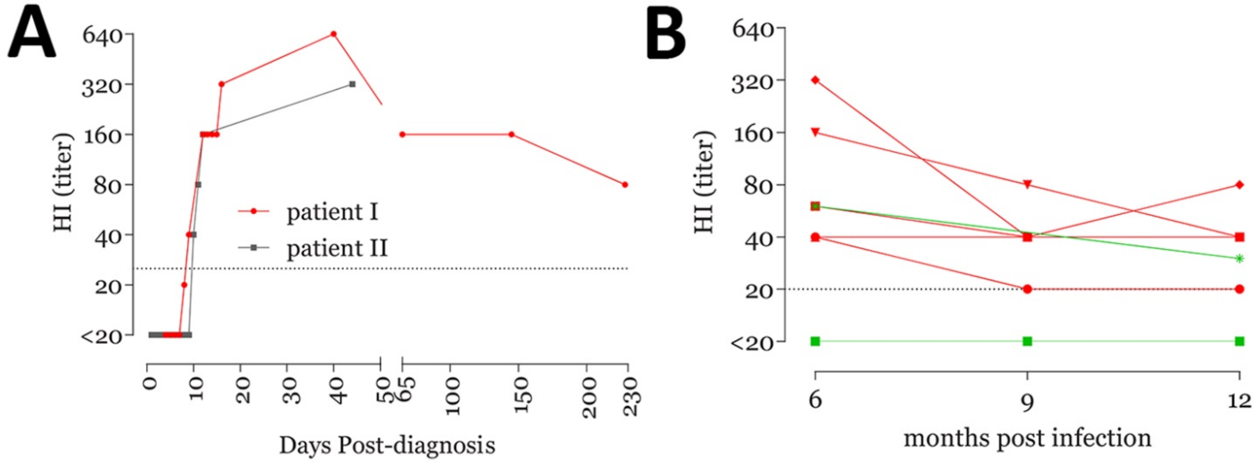

Appendix Figure 5. Kinetics of HI antibody responses in PCR-confirmed MERS patients. HI antibody titers in (A) two acute to convalescent phase patients and in (B) six severe (red) and five mild (green) MERS-CoV patients six to twelve months post-infection.

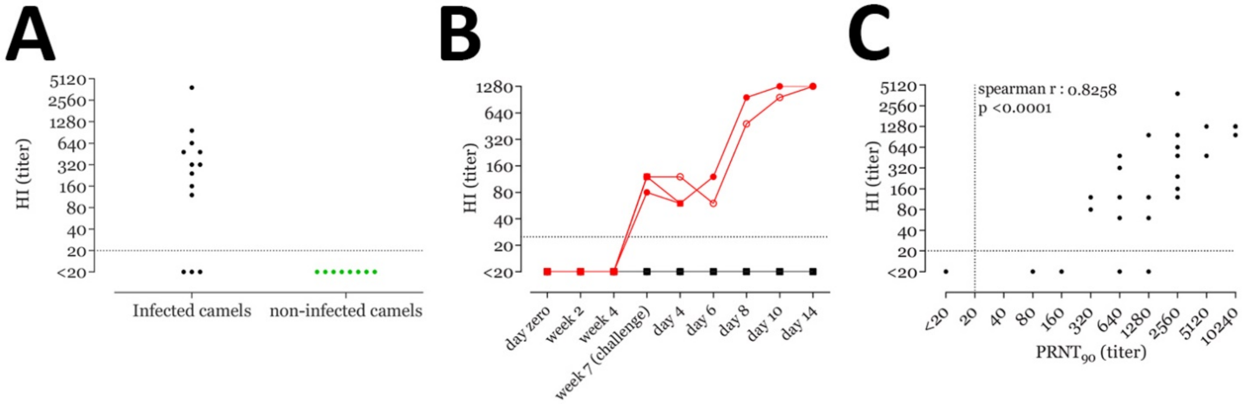

Appendix Figure 6. MERS-CoV hemagglutination inhibition (HI) assay for MERS-CoV dromedary camel diagnostics. A) Validation of the specificity of the HI assay for the detection of MERS-CoV specific antibodies in the sera of MERS-CoV-infected (black) and naïve (green) dromedary camels. B) Kinetics of HI antibody responses in dromedary camels following vaccination (red) and infection (black). C) Correlation between neutralizing and HI antibody responses following MERS-CoV infection or vaccination in dromedaries. $PRNT_{90}$, 90% reduction in plaque reduction neutralization test.
